# Risk factors for territorial spreading of SARS-CoV-2 in North-eastern Italy

**DOI:** 10.1038/s41598-022-05368-8

**Published:** 2022-02-09

**Authors:** Ettore Bidoli, Federica Toffolutti, Stefania Del Zotto, Diego Serraino

**Affiliations:** 1 Unit of Cancer Epidemiology, Centro di Riferimento Oncologico di Aviano (CRO), IRCCS, via Gallini 2, 33081 Aviano, PN Italy; 2 Azienda Regionale di Coordinamento per la Salute (ARCS), Udine, Italy

**Keywords:** Medical research, Epidemiology, SARS-CoV-2

## Abstract

The impact of specific risk factors for SARS-CoV-2 infection spread was investigated among the 215 municipalities in north-eastern Italy. SARS-CoV-2 incidence was gathered fortnightly since April 1, 2020 (21 consecutive periods) to depict three indicators of virus spreading from hierarchical Bayesian maps. Eight explanatory features of the municipalities were obtained from official databases (urbanicity, population density, active population on total, hosting schools or nursing homes, proportion of commuting workers or students, and percent of > 75 years population on total). Multivariate Odds Ratios (ORs), and corresponding 95% Confidence Intervals (CIs), quantified the associations between municipality features and virus spreading. The municipalities hosting nursing homes showed an excess of positive tested cases (OR = 2.61, ever versus never, 95% CI 1.37;4.98), and displayed repeated significant excesses: OR = 5.43, 3–4 times versus 0 (95% CI 1.98;14.87) and OR = 6.10, > 5 times versus 0 (95% CI 1.60;23.30). Municipalities with an active population > 50% were linked to a unique statistical excess of cases (OR = 3.06, 1 time versus 0, 95% CI 1.43;6.57) and were inversely related to repeated statistically significant excesses (OR = 0.25, > 5 times versus 0; 95% CI 0.06;0.98). We highlighted specific municipality features that give clues about SARS-CoV-2 prevention.

## Introduction

The corona virus disease-19 (COVID-19) pandemic, caused by infection with severe acute respiratory syndrome coronavirus 2 (SARS-CoV-2) upset the world. In the 1.2-million-inhabitant Friuli Venezia Giulia (FVG) region, northeastern Italy, the first individual with confirmed SARS-CoV-2 infection was detected on February 29, 2020 after a contact with another case during a local agricultural engineering congress^1^.

Three studies conducted during the initial phase of the COVID-19 outbreak identified individual factors associated with the local spread of SARS-CoV-2. A cross-sectional study^2^ examined and mapped from March to April 2020 the cumulative incidence and health conditions of infected individuals revealing that 25% of them were hosted in retirement homes. In the same time, the fastest spread of the infection together with the highest frequency of cases were observed in the the city of Trieste (200,000 inhabitants), i.e., FVG major urban area while a reduced transmission of the virus was observed in less densely populated areas. Two retrospective cohort studies showed the greatest risk of hospitalization or death in males, elderly and in individuals with comorbidities^3,4^, whereas no evidence emerged for an increased mortality in guests of nursing homes^4^. Similar patterns were observed worldwide during the initial period of virus spreading^5^, while lockdown policies implemented to avoid or to slow down the viral spread (e.g., mandatory facial covering, home quarantine, social distancing, regional border closures, travel bans, and school or non-essential business restrictions) have modified the epidemiological patterns observed in the initial period of the local pandemic.

Prevention approach by case isolation, until contagion becomes unlikely, is an important line of attack to reduce SARS-CoV-2 spread. To implement this type of prevention, it is crucial to find out if and how frequently specific municipalities had a statistically significant link to excesses of infected cases. To this aim, we described three indicators of SARS-CoV-2 spread at municipality level from 21 consecutive fortnightly independent cross-sectional hierarchical Bayesian maps, and quantified their association with eight specific explanatory municipality features.

To systematically address this issue, we took advantage of the availability of computerized data of confirmed SARS-CoV-2 cases aggregated at municipality (N = 215) level in the FVG region, and of regional or national official data of explanatory features. All data were examined fortnightly 21 times since April 1st, 2020.

## Materials and methods

An observational, ecological study was conducted using all confirmed SARS-CoV-2 cases in the FVG region from April 1, 2020 to February 1, 2021. We extracted all data from official publicly available databases and depicted three indicators of SARS-CoV-2 spreading using hierarchical Bayesian disease-mapping techniques.

### Official data sources

A. Data related to cases were originally gathered by the 18 territorial Prevention Departments of the FVG region, and successively coded, validated, and registered by the Regional Health Center Directorate, and made publicly available weekly through the Regional Department of Civil Protection (RDCP) website^6^ by means of a comma delimited text file. Data were disentangled at municipality level (N = 215), the smallest Italian administrative area, without reference to age or sex of SARS-CoV-2 cases. The header of the text file reported the variable names only in the Italian language. Three relevant variables for the study were extracted from the file: 1) the numeric code of each municipality of the FVG region (reported as “ISTAT”, i.e. the code defined by the Italian National Institute of Statistics); 2) the calendar date of the data collection (reported as “Data”); and 3) the cumulative number of alive persons confirmed positive and still without a negative SARS-CoV-2 test (reported as “Attualmente positivi”). It should be noted that the exact date of diagnosis of the cases was not reported in the file. It has been described that patients with mild-to-moderate SARS-CoV-2 remained infectious no longer that 10 days after symptom onset^7^, while most patients with severe-to-critical SARS-CoV-2 remained infectious no longer that 20 days, except immunocompromized patients who remained infectious beyond 20 days. The severe-to-critical patients were those requiring hospitalization, intensive care, or ventilation support (e.g. patients that remained in strict isolation). Given these assumptions and the type of available data, we chose a priori to examine data fortnightly (the first and 15th day of each month, consecutively for 21 times since April 1st, 2020), empirically assuming that a 2-week lag (precisely: 15.3 ± 1.4 days) between analyses was adequate to limit the number of overlapping cases in two consecutive periods.
B. The explanatory features of the municipalities were gathered from three different open-access data sources that are routinely collected. In order to reduce as much as possible the uncertainty of mixing different kind of data sources, we accessed only official, reliable, and well-referenced public data at Italian institutional level. Features have always been recorded in two groups due to the low number of municipalities in FVG region (N = 215). Features consisting of continuous data were split into two groups by setting the median as the cut-off. The feature: Municipalities hosting nursing homes (coded: no/yes) was directly provided by the FVG region^8^, last update, mid-February 2019. The other features were downloaded from the Italian National Institute of Statistics (ISTAT)^9^. In particular: 1) The percentage of the population aged between 20 and 59 years to total population, used as a generic indicator of people with potential and generic mobility (recoded as < 50% and ≥ 50%); 2) the percentage of the population aged above 75 years to the total population, used as an indicator of population at high risk of Covid-19 infection (recoded as < 14% and ≥ 14%); 3) the percentage of commuting students (recoded as < 14.4%. and ≥ 14.4%) and the percentage of commuting workers (recoded as < 50%. and ≥ 50%) to the total population, both used as indicators of people crossing the border of their municipality of residence on daily basis—these two indicators were released during the 2011 Italian national census; 4) the population density (recoded as < 100 and ≥ 100 inhabitants/km^2^), and the urban–rural gradient both used as indicators of high risk municipalities; and 5) the municipalities hosting schools of any grade^10^.
C. The number of resident FVG population (around 1,200,000 inhabitants) by municipality was abstracted from the same publicly available database of the cases (i.e. RDCP data source).

### Indicators of the SARS-CoV-2 infection spreading

The indicators of SARS-CoV-2 spreading were estimated by computing 21 consecutive fortnightly maps, including the number of confirmed positive tested cases. We used a hierarchical Bayesian Conditional Autoregressive (CAR) model^11^ to produce stabilized estimates of the Relative Risks (RR) of COVID-19 infections for each municipality and their corresponding Posterior Probabilities (PP). The CAR model was fitted fortnightly by means of WinBUGS (v. 1.41), a public domain package for Bayesian inference using Markov Chain Monte Carlo (MCMC) methods. For each 14-day period the expected numbers of cases for each municipality were computed using the sum of cases as the reference. Area-specific heterogeneity was accounted for by considering two-component random effects: 1) an unstructured effect (uncorrelated heterogeneity); and 2) a group of neighboring random effects (correlated heterogeneity). The model parameters were estimated using Gibbs sampling. Model fitting was carried out using three separate Markov chains starting from different initial values. The first 60,000 samples from each chain were discarded as burn-in, and the following 40,000 iterations were sampled. Convergence was checked by visual inspection of the time series samples plots and by examination of four diagnostic indicators (Geweke, Gelman-Rubin, Raferty-Lewis, and Heidelberg-Welch diagnostics) produced by the coda package^12^. RRs and PPs were graphically displayed using two separate maps at municipality level. In one map, RR values were grouped in five categories (< 0.90, 0.90–0.94, 0.95–1.04, 1.05–1.09, and ≥ 1.10) with a color scheme of red tonalities. In a second map, PPs were grouped in five categories (< 2.5%, 2.5–9%, 10–89%, 90–97.5%, ≥ 97.5%) with gray tonalities. The boundaries of the four provinces of FVG region (i.e. administrative areas with one main urban center) were added to the maps.

In order to calculate the three indicators of SARS-CoV-2 spreading, we exploited the whole set of 21 consecutive maps to group municipalities a priori according to the characteristics of the statistically significant excesses of the RRs. Specifically, in each municipality and for every 21 fortnightly maps we counted the number of times risk of SARS-CoV-2 presented a statistically significant excess (i.e., the times RR was significantly above 1 and with a PP ≥ 97.5%). In other words, this count of significant excesses allowed to link cases to specific municipalities repeatedly across examined periods and to measure their association with the studied features. Three indicators were obtained from the counts: 1) Statistically significant excesses (coded as “Never” for 0 excesses out of 21 maps, “Ever” for at least one excess in 21 maps) used to detect directly high risk municipalities; 2) peak periods (coded as “Never” for 0 excesses in 21 maps), “First peak” for an excess exclusively during the first peak (between 2020/04/01 and 2020/07/01), “Second peak” for an excess exclusively during the second peak (after 2020/07/01), and “Both” for at least one excess in both peaks) used as a sensitivity analysis of the study across peaks; and 3) number of repeated statistically significant excesses (Never, 1, 2, 3–4, and ≥ 5) used to measure the gravity of the virus spread by means of a frequency in each municipality across 21 maps.

### Statistical analyses

We computed crude rates (CR) per 10,000 (number of cases / population per 10,000) and counted the number of municipalities that displayed statistically significant excesses (RR > 1 with a PP ≥ 97.5%). Odds ratios (OR), and their corresponding 95% confidence intervals (CI), were computed using unconditional multiple logistic regression models^13^ to quantify the association between the explanatory municipalities features and the COVID-19 spreading indicators. A p-value < 0.05 was considered statistically significant. All statistical analyses were carried out using SAS (version 9.4 SAS Institute, Cary, NC, USA).

## Results

**Figure 1:**
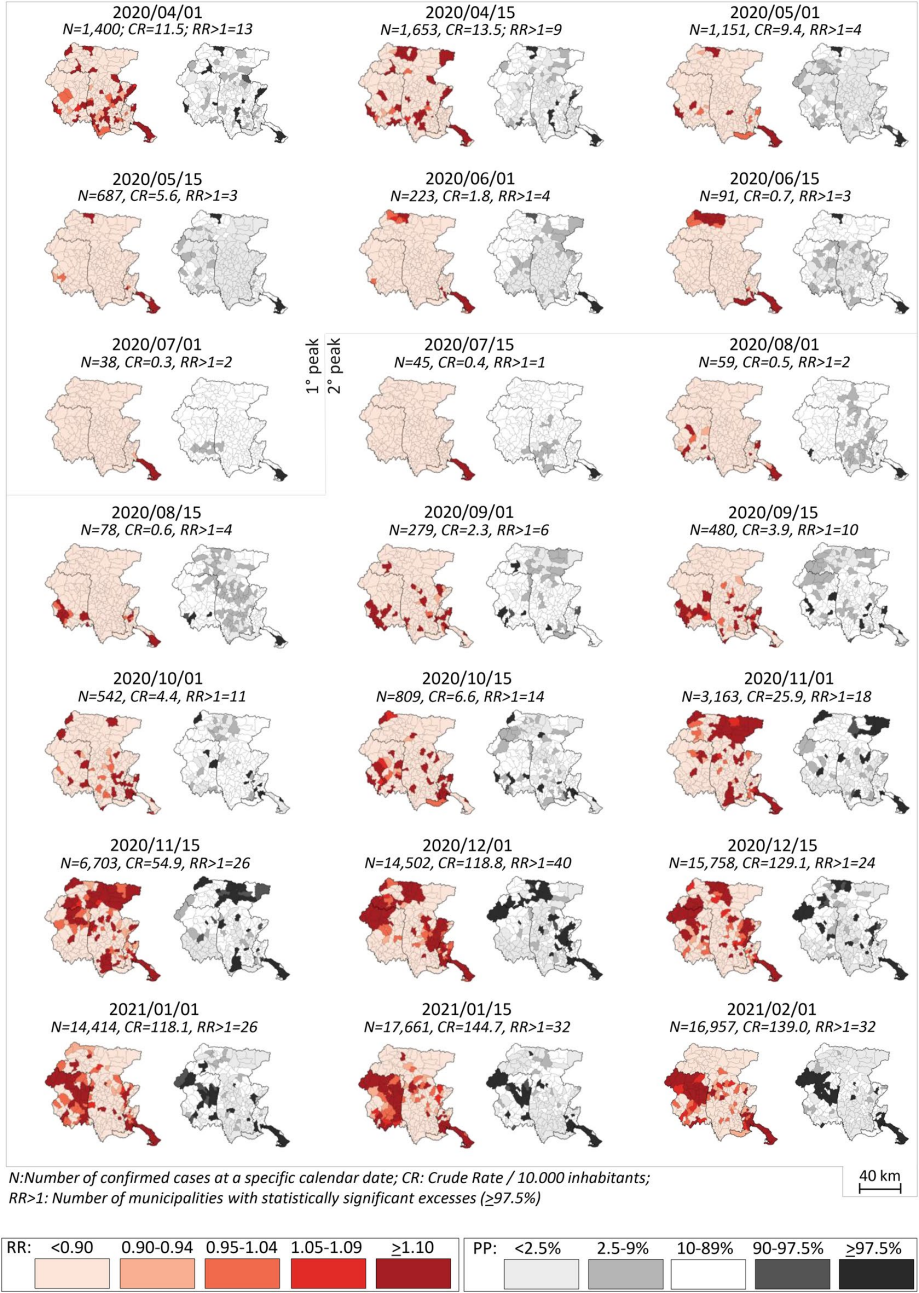
Twenty-one fortnightly consecutive hierarchical Bayesian maps of Relative Risks (RR), and corresponding Posterior Probabilities (PP), for confirmed SARS-CoV-2 cases at a specific calendar date; April 1, 2020 to February 1, 2021. Friuli Venezia Giulia Region, Italy.

Figure 1 displays the spatial distribution and the descriptive indicators (number of cases and crude rates) associated with confirmed SARS-CoV-2 positive cases among the 215 municipalities of FVG region, during 21 consecutive fortnightly periods from April 1, 2020 to February 1, 2021. The first map showed that the number of confirmed SARS-CoV-2 positive cases was 1400 corresponding to a CR of 11.5/10,000 inhabitants. Cases increased only during the following 14-days (N = 1653, CR = 13.5) to decline continuously up to July 1, 2020 (N = 38, CR = 0.3). Afterwards, the number of cases increased up to January 15, 2021 (N = 17,661, CR = 144.7) and subsequently plateaued. Based on these observations, we decided to split the study period into two peaks (one: from April 1, 2020 to July 1, 2020, and the second peak thereafter). A tenfold increase in the number of cases occurred between the tip of the first peak and that of the second peak (1653 vs. 17,661 respectively).

The varying spatial distribution of cases highlighted three main patterns (Fig. 1). Firstly, in the lower right corner of the maps, five municipalities tended to cluster around the capital city of FVG region. Secondly, from November 1, 2020 part of the excesses seemed to move counterwise from North-east in the upper right corner of the map) to South-west (lower left). Thirdly, when jointly examining the 21 maps, 104 municipalities (out of 215) never displayed a statistically significant excess.

Table 1 gives the distribution of the explanatory features of the 215 municipalities of the FVG region (i.e. population density, urban–rural gradient, hosting of nursing homes, percent of ≥ 75 years population to total population, percent of 20–59 years population to total population, percent of commuting workers to total population, hosting schools of any grade, and percent of commuting students to total population) according to the three dimensions of the COVID-19 pandemic spread deducted from the 21 fortnightly Bayesian maps. Municipalities hosting nursing homes, hosting schools of any grade, and with a percentage of 20–59 years population higher or equal than 50%, were associated (p-value < 0.05) with all the three dimensions of COVID-19 pandemic spread.

**Table 1:** Distribution of eight features of the 215 municipalities of the FVG region according to three indicators of SARS-COV-2 spread. FVG region, from April 1, 2020 to February 1, 2021.

Indicators of SARS-COV-2 spread	Municipality features																															
	Population density (inhabitants/km2)				Urban rural gradient				Hosting nursing homes				% of > 75years population*				% of 20–59 years population*				Percent of commuting workers (2011 census)*				Hosting schools of any grade				% of commuting students (2011 census)*			
	< 100		>100		Rural		Urban		No		Yes		< 14%		> 14%		< 50%		>50%		< 50%		>50%		No		Si		< 14.4%		> 14.4%	
	N	(%)	N	(%)	N	(%)	N	(%)	N	(%)	N	(%)	N	(%)	N	(%)	N	(%)	N	(%)	N	(%)	N	(%)	N	(%)	N	(%)	N	(%)	N	(%)
**Statistically significant excesses**																																
Never	54	(52.4)	50	(44.6)	79	(52.3)	25	(39.1)	82	(56.2)	22	(31.9)	53	(46.9)	51	(50.0)	61	(55.0)	43	(41.3)	54	(50.0)	50	(46.7)	76	(55.1)	28	(36.4)	57	(52.8)	47	(43.9)
Ever	49	(47.6)	62	(55.4)	72	(47.7)	39	(60.9)	64	(43.8)	47	(68.1)	60	(53.1)	51	(50.0)	50	(45.1)	61	(58.7)	54	(50.0)	57	(53.3)	62	(44.9)	49	(63.6)	51	(47.2)	60	(56.1)
X^2^_1_	1.30; *p* = 0.25				3.16; *p* = 0.08				11.06; *p* < 0.001				0.21; *p* = 0.65				3.98; *p* = 0.04				0.23; *p* = 0.63				6.93; *p* = 0.01					1.69; *p* = 0.19		
**Peak period**																																
Never	54	(52.4)	50	(44.6)	79	(52.3)	25	(39.1)	82	(56.2)	22	(31.9)	53	(46.9)	51	(50.0)	61	(55.0)	43	(41.3)	54	(50.0)	50	(46.7)	76	(55.1)	28	(36.4)	57	(52.8)	47	(43.9)
First peak	3	(2.9)	1	(0.9)	4	(2.7)	0	(-)	3	(2.1)	1	(1.5)	1	(0.9)	3	(2.9)	2	(1.8)	2	(1.9)	2	(1.9)	2	(1.9)	3	(2.2)	1	(1.3)	2	(1.9)	2	(1.9)
Second peak	41	(43.2)	53	(51.5)	60	(43.2)	34	(53.1)	55	(40.2)	39	(63.9)	55	(48.7)	39	(38.2)	38	(38.4)	56	(56.6)	43	(39.8)	51	(47.7)	53	(38.4)	41	(53.3)	44	(44.7)	50	(46.7)
Both	5	(4.9)	8	(7.1)	8	(5.3)	5	(7.8)	6	(4.1)	7	(10.1)	4	(3.5)	9	(8.8)	10	(9.0)	3	(2.9)	9	(8.3)	4	(3.7)	6	(4.4)	7	(9.1)	5	(4.6)	8	(7.5)
X^2^_3_	3.01; *p* = 0.39				5.64; *p* = 0.13				12.43; *p* = 0.01				5.14; *p* = 0.16				10.11; *p* = 0.02				2.75; *p* = 0.43				8.11; *p* = 0.04				2.03; *p* = 0.57			
**Number of statistically significant excesses**																																
0	54	(52.4)	50	(44.6)	79	(52.3)	25	(39.1)	82	(56.2)	22	(31.9)	53	(46.9)	51	(50.0)	61	(55.0)	43	(41.3)	54	(50.0)	50	(46.7)	76	(55.1)	28	(36.4)	57	(52.8)	47	(43.9)
1	20	(19.4)	27	(24.1)	36	(23.8)	11	(17.2)	31	(21.2)	16	(23.2)	28	(24.8)	19	(18.6)	16	(14.4)	31	(29.8)	22	(20.4)	25	(23.4)	29	(21.0)	18	(23.4)	19	(17.6)	28	(26.2)
2	14	(13.6)	12	(10.7)	18	(11.9)	8	(12.5)	20	(13.7)	6	(8.7)	12	(10.6)	14	(13.7)	12	(10.8)	14	(13.5)	13	(12.0)	13	(12.5)	16	(11.6)	10	(13.0)	14	(13.0)	12	(11.2)
3–4	12	(11.7)	12	(10.7)	13	(8.6)	11	(17.1)	9	(6.2)	15	(21.7)	14	(12.4)	10	(9.8)	12	(10.8)	12	(11.5)	13	(12.0)	11	(10.3)	12	(8.7)	12	(15.6)	10	(9.3)	14	(13.1)
> 5	3	(2.9)	11	(9.8)	5	(3.3)	9	(14.1)	4	(2.7)	10	(14.5)	6	(5.3)	8	(7.8)	10	(9.0)	4	(3.9)	6	(5.6)	8	(7.5)	5	(3.6)	9	(11.7)	8	(7.4)	6	(5.6)
X^2^_4_	5.55; *p* = 0.24				13.50; *p* = 0.01				26.88; *p* < 0.001				2.31; *p* = 0.68				10.41; *p* = 0.03				0.79; *p* = 0.94				10.82; *p* = 0.03				3.79; *p* = 0.44			

*To total population

Table 2 gives the univariate ORs of the studied features of the municipalities according to the three dimensions of the COVID-19 spread. Municipalities hosting nursing homes showed a statistically significant excess of COVID-19 cases (OR = 2.74 for ever versus never; 95% CI: 1.50–5.00) with a repeated number of statistical excesses: OR = 6.21, for 3–4 times vs. 0, in 21 fortnightly periods (95% CI: 2.40–16.08) and OR = 9.32, for ≥ 5 times vs. 0 (95% CI: 2.67–32.57). Municipalities with a percentage of a population of 20–59 years above or equal 50% displayed a statistically significant excess of COVID-19 positive cases (OR = 1.73 for ever versus never; 95% CI: 1.01–2.97) linked to a unique statistical excess (OR = 2.75, for one time vs. 0; 95% CI: 1.34–5.64). Municipalities that hosted schools of any grade showed a repeated number of excesses: OR = 2.71, for 3–4 times vs. 0 (95% CI: 1.09–6.74) and OR = 4.89, for ≥ 5 times vs. 0 (95% CI: 1.51–15.84). Population density above or equal 100 inhabitants/km^2^ was associated with a repeated number of statistically significant excesses of COVID-19 (OR = 3.96, for ≥ 5 times vs. 0; 95% CI: 1.04–15.02). Similarly, urbanicity was associated with a repeated number of statistically significant excesses of COVID-19 (OR = 5.69, for ≥ 5 times vs. 0; 95% CI: 1.74–15.55). The first peak was merely associated with four municipalities excesses, which indicated a low power of the analyses. Although not statistically significant, the risk pattern of the features did not diverge between the two peaks.

**Table 2:** Univariate^‡^ odds ratios (OR) of eight features of the 215 municipalities of FVG region, and corresponding 95% confidence intervals (CIs), according to three indicators of SARS-COV-2 spread. FVG region, from April 1, 2020 to February 1, 2021.

Indicators of SARS-COV-2 spread	Municipality features															
	Population density (inhabitants/km2): (> 100 vs. < 100)		Urban vs. rural gradient		Hosting nursing homes (Yes vs. No)		% of >75years population* (> 14 vs. < 14)		% of 20-59 years population* (> 50 vs. < 50)		Percent of commuting workers (2011 census)* (> 50 vs. < 50)		Hosting school of any grade (Yes vs. no)		% of commuting students (2011 census)* (> 14.4 vs. < 14.4)	
	OR	(95% CI)	OR	(95% CI)	OR	(95% CI)	OR	(95% CI)	OR	(95% CI)	OR	(95% CI)	OR	(95% CI)	OR	(95% CI)
**Statistically significant excesses**																
Never	1^†^		1 +		1 +		1 +		1 +		1 +		1 +		1+	
Ever	1.37	(0.80; 2.34)	1.71	(0.94; 3.10)	2.74	(1.50; 5.00)	0.88	(0.52; 1.51)	1.73	(1.01; 2.97)	1.14	(0.67; 1,95)	2.15	(1.21; 3.80)	1.43	(0.83; 2.44)
**Peak period**																
Never	1^†^		1 +		1 +		1 +		1 +		1 +		1 +		1 +	
First peak	0.36	(0.04; 3.58)	-	-	1.24	(0.12; 12.54)	3.12	(0.31; 30.96)	1.42	(0.19; 10.47)	1.08	(0.15; 7.96)	0.91	(0.09; 9.06)	1.21	(0.17; 8.94)
Second peak	1.40	(0.80; 2.45)	1.79	(0.97; 3.32)	2.64	(1.42; 4.93)	0.74	(0.42; 1.29)	2.09	(1.19; 3.69)	1.28	(0.73; 2.24)	2.10	(1.16; 3.81)	1.38	(0.79; 2.41)
Both	1.73	(0.53; 5.63)	1.98	(0.59; 6.59)	4.35	(1.33; 14.26)	2.34	(0.68; 8.07)	0.43	(0.11; 1.64)	0.48	(0.14; 1.66)	3.17	(0.98; 10.23)	1.94	(0.60; 6.33)
**Number of statistically significant excesses**																
0	1^†^		1 +		1 +		1 +		1 +		1 +		1 +		1 +	
1	1.46	(0.73; 2.92)	0.97	(0.43; 2.17)	1.92	(0.90; 4.14)	0.71	(0.35; 1.42)	2.75	(1.34; 5.64)	1.23	(0.62; 2.45)	1.69	(0.81; 3.50)	1.79	(0.89; 3.60)
2	0.93	(0.39; 2.19)	1.40	(0.55; 3.62)	1.12	(0.40; 3.12)	1.21	(0.51; 2.87)	1.66	(0.70; 3.93)	1.08	(0.46; 2.55)	1.70	(0.69; 4.18)	1.04	(0.44; 2.46)
3–4	1.08	(0.44; 2.62)	2.67	(0.07; 6.71)	6.21	(2.40; 16.08)	0.74	(0.30; 1.82)	1.42	(0.58; 3.46)	0.91	(0.38; 2.23)	2.71	(1.09; 6.74)	1.70	(0.69; 4.17)
> 5	3.96	(1.04; 15.02)	5.69	(1.74; 15.55)	9.32	(2.67; 32.57)	1.39	(0.45; 4.27)	0.57	(0.17; 1.93)	1.44	(0.47; 4.44)	4.89	(1.51; 15.84)	0.91	(0.30; 2.81)

*To total population.

^†^Reference category.

^‡^Estimates from logistic regression.

These associations were further examined by means of a multivariate model that included the five statistically significant explanatory features emerged in the univariate model (Table 3). Hosting nursing homes and the proportion of 20–59 years population on total were confirmed as associated with COVID-19 spreading. In particular, municipalities hosting nursing homes showed a statistically significant excess of SARS-CoV-2 positive cases (OR = 2.61 for ever versus never; 95% CI: 1.37–4.98), and displayed repeated significant excesses: OR = 5.43 for 3–4 times vs. 0 (95% CI: 1.98–14.87) and OR = 6.10, for ≥ 5 times vs. 0 (95% CI: 1.60–23.30). Municipalities with a proportion of 20–59 years population ≥ 50% were linked to a unique number of statistical excesses in 21 periods (OR = 3.06; 95% CI: 1.43–6.57), and they were inversely related to repeated statistically significant excesses of SARS-CoV-2 positive cases (OR = 0.25, for ≥ 5 times vs. 0; 95% CI: 0.06–0.98). Finally, the associations observed in the univariate analysis for population density, urbanicity, and hosting schools of any grade lost their effect in the multivariate model.

**Table 3:** Multivariate^‡^ odds ratios (OR) of five features of the 215 municipalities of FVG region, and corresponding 95% confidence intervals (CIs), according to three indicators of SARS-COV-2 spread. FVG region, from April 1, 2020 to February 1, 2021.

Indicators of SARS-COV-2 spread	Municipality features									
	Population density (inhabitants/km2): (>100 vs. < 100)		Urban vs. rural gradient		Hosting nursing homes (Yes vs. No)		% of 20-59 years population* (>50 vs. < 50)		Hosting schools of any grade (Yes vs. No)	
	OR	(95% CI)	OR	(95% CI)	OR	(95% CI)	OR	(95% CI)	OR	(95% CI)
**Statistically significant excesses**										
Never	1^†^		1^†^		1^†^		1^†^		1^†^	
Ever	0.75	(0.36; 1.58)	0.94	(0.44; 2.02)	2.61	(1.37; 4.98)	1.62	(0.90; 2.89)	1.52	(0.75; 3.10)
**Peak period**										
Never	1^†^		1^†^		1^†^		1^†^		1^†^	
First peak	0.43	(0.04; 5.17)	-	-	2.14	(0.21; 22.12)	2.05	(0.27; 15.29)	1.70	(0.15; 19.80)
Second peak	0.72	(0.33; 1.57)	0.94	(0.42; 2.08)	2.48	(1.27; 4.85)	1.93	(1.05; 3.56)	1.46	(0.70; 3.06)
Both	1.22	(0.26; 5.66)	1.38	(0.34; 5.65)	3.89	(1.07; 14.17)	0.30	(0.07; 1.31)	1.98	(0.48; 8.12)
**Number of statistically significant excesses**										
0	1^†^		1^†^		1^†^		1^†^		1^†^	
1	1.02	(0.42; 2.50)	0.46	(0.17; 1.25)	2.15	(0.94; 4.90)	3.06	(1.43; 6.57)	1.75	(0.70; 4.40)
2	0.50	(0.15; 1.74)	0.96	(0.29; 3.13)	1.02	(0.34; 3.00)	1.55	(0.61; 3.93)	1.70	(0.57; 5.06)
3–4	0.23	(0.05; 1.10)	1.43	(0.42; 4.83)	5.43	(1.98; 14.87)	1.03	(0.37; 2.87)	1.11	(0.35; 3.50)
> 5	2.27	(0.35; 14.95)	4.23	(0.96; 18.61)	6.10	(1.60; 23.30)	0.25	(0.06; 0.98)	1.34	(0.33; 5.42)

*To total population.

^†^Reference category.

^‡^Estimates from logistic regression adjusted mutually for the above features, when appropriate.

## Discussion

Our observational study, which was based on all confirmed SARS-CoV-2 positive cases diagnosed in a well-defined population during 21 fortnightly periods, with data centrally validated since the beginning of the pandemic, showed that municipalities hosting nursing homes besides displaying a statistically significant excess of positive tested cases were also at risk of at least 3–4 repeated excesses. By contrast, municipalities with the higher proportion of a population aged 20–59 years to total (i.e., the population in the most active age) were linked to a unique statistically significant excess in the 21 fortnightly periods, and they were inversely and significantly associated with five or more repeated excesses of SARS-CoV-2 positive cases. Finally, population density, urbanicity and hosting schools of any grade lost their effect when standardized for the aforementioned features of the municipalities (i.e., hosting nursing homes or a higher proportion of active population).

These results, which are broadly consistent with the findings from other investigations, add further information on the features associated to sporadic or repeated excesses of the virus spreading and on the features that may have been contrasted by lockdown policies. The observed pattern gives clues to prevent spread of SARS-CoV-2 infection in municipalities with well-defined features. Several plausible biological mechanisms linked to population demography^5^ and to specific SARS-CoV-2 infection risk factors^14^ may elucidate the associations measured by our study.

Municipalities hosting nursing homes showed an excess of SARS-CoV-2 positive cases along with repeated significant excesses (of at least 3–4 times over the 21 fortnightly periods). Assuming that the excesses observed in our study were partially or almost totally linked to the nursing homes located within these municipalities, our observation is consistent with previous papers published in a number of countries^15,16^. In particular, in the US, the vulnerability of nursing homes in relation to SARS-CoV-2 infection^17^ has been defined as the “Perfect Storm” ^18^. Moreover, our observation of repeated excesses in the same municipalities of FVG suggested, firstly, that once the virus entered the nursing homes, it was difficult to eradicate it in a short period of time and, secondly, that lockdown policies regarding access of relatives or external visitors unaffected the virus infection course. It is well-known that nursing homes accommodate vulnerable persons into shared living or close quarters or communal spaces. These persons generally presented chronic comorbidities (such as, diabetes, cardiovascular diseases, chronic respiratory diseases, cerebrovascular diseases, malignancies or dementia) and various degrees of disability, which may hinder preventive health measures, such as personal hand washing^19^. An alternate explanation of the pattern observed is a higher rate of SARS-CoV-2 testing in nursing homes than in the general population; however, this hypothesis should be tested with a different study design.

Municipalities with a ≥ 50% proportion of 20–59-year old population were linked to a unique statistically significant excess of tested cases for SARS-CoV-2 over the 21 periods, but they were also inversely related to ≥ 5 statistically significant excesses. The age range studied is a proxy of the most active portion of the population that is also linked to population mobility^20^. This direct association has been previously reported in nearly 52 countries^20,21^ together with the benefits of the reduction in mobility in the virus transmission^21^. Thus, in FVG region, lockdown policies should have limited the mobility patterns and reduced transmissibility of the virus. However, it seems that the mobility containment unaffected sporadic clusters temporally limited to 14-days while these measures avoided statistically significant repeated excesses in the municipalities with a higher proportion of active population. Moreover, the excesses limited to a 14-day period suggested that some kind of isolation or quarantine may have limited the further spreading of the infection.

In our study, municipalities hosting schools were associated with repeated significant excesses (of at least 3–4 times or more in the 21 periods examined). After adjusting for all significant features mututally, the hosting schools feature lost its effects. There is limited evidence that schools had a relevant role in SARS-CoV-2 transmission in the general population^22–24^, although indications that community transmission can be imported into the school setting has been described^25^. Evidence of increased risk of reported SARS-CoV-2 infection and COVID-19 outcomes has been reported among adults living with children during the second peak. However, this did not translate into a materially increased risk of covid-19 mortality, and absolute increases in risk were small^26^. In any case, further studies are needed to elucidate the role of schools in the ongoing pandemic in the light of potentially low social distancing between scholars (during lessons or travel from/to school).

By univariate analysis, municipalities with a population density higher than or equal to 100 inhabitants/km^2^ (vs. lower than 100), or classified as urban (vs. rural) showed a statistically significant association only between repeated excesses (higher or equal than 5 times) of SARS-CoV-2 positive cases. This observation was not confirmed in the multivariate model. It is known that SARS-CoV-2 is an aerial pathogen, and population density or urbanicity might have played a significant role in the acceleration of transmission, together with overcrowding influx following social events^27–29^. Our result suggested that lockdown policies contained the natural spreading of the virus.

Several strengths of this investigation deserve attention. First, the present study took advantage of a regional database containing information about SARS-CoV-2 infection of all residents. Second, this database met Italian standards for validation, high-quality, and comparability of the methodology of registration, thus included in the national COVID-19 database. Third, the features of the municipalities were obtained from official open-access data sources that are routinely collected for national censuses. Fourth, we assumed the residential location of the participants as a proxy of individual exposure. Our study population was relatively stable, with nearly 93% of the FVG population living in their area of residence for more than 15 years^30^.

Conversely, the study suffered from some worth noting limitations, some common to other observational studies. First the lack of availability of individual level data such as age, sex, date of diagnosis or time spent in the municipality of residence avoided intragroup comparison and the computation of spatio-temporal models. However, in our study the lack of this type of information should have only flattened the risk estimates if random with respect to virus spreading. Second, the data were gathered territorially by multiple teams and no information was available about the type of population tested (i.e. on voluntary basis, screened or symptomatic). Third, undiagnosed patients (i.e. asymptomatic, misdiagnosed, or dead individuals) might flatten our estimates; however, due to the mode of virus transmission, it is unlikely that these cloaked positive individuals had different risk factors than those reported in the official data. Thus, although the maps represented strictly infected patients with a positive test, they can give clues about the whole pattern of infection. Fourth, lockdown policies should have influenced the spread pattern of SARS-CoV-2, though whether the policies were homogeneously applied in the whole FVG region is an unanswered question.

In conclusion, while waiting for the global vaccination against COVID-19, the best way to prevent infection is by case identification and isolation until contagion becomes unlikely. However, the implementation of centered measures of prevention require knowledge of the routes of transmission. We implemented key parameters able to describe the virus spread at municipality level and quantified their association with several explanatory features linked to municipalities. These findings give clues for better preventing the SARS-CoV-2 pandemic at local level.

## Data Availability

The datasets used and/or analyzed during the current study are available from the first author or by download of the dataset from the reported links.
